# After the madhouses: the emotional politics of psychiatry and community care in the UK tabloid press 1980–1995

**DOI:** 10.1136/medhum-2020-012117

**Published:** 2021-08-20

**Authors:** Leah Sidi

**Affiliations:** School of European Languages and Cultures, UCL, London WC1E 6BT, UK

**Keywords:** psychiatry, popular media, medical humanities

## Abstract

The deinstitutionalisation of mental hospital patients made its way into UK statutory law in 1990 in the form of the NHS and the Community Care Act. The Act ushered in the final stage of asylum closures moving the responsibility for the long-term care of mentally ill individuals out of the NHS and into the hands of local authorities. This article examines the reaction to the passing of the Act in two major tabloid presses, *The Sun* and *The Daily Mirror*, in order to reveal how community care changed the emotional terrain of tabloid storytelling on mental health. Reviewing an archive of 15 years of tabloid reporting on mental illness, I argue that the generation of ‘objects of feeling’ in the tabloid media is dependent on the availability of recognisable and stable symbols. Tabloid reporting of mental illness before 1990 reveals the dominance of the image of the asylum in popular understandings of mental illness. Here the asylum is used to generate objects of hatred and disgust for the reader, even as it performs a straightforward othering and distancing function. In these articles, the image of the asylum and its implicit separation of different types of madness into categories also do normative gender work as mental illness is represented along predictable gendered stereotypes. By performing the abolition of asylums, the 1990 Act appears to have triggered a dislodging of these narrative norms in the tabloid press. After 1990, ‘asylum stories’ are replaced with ‘community care stories’ which contain more contradictory and confusing clusters of feeling. These stories rest less heavily on gendered binaries while also demonstrating a near-frantic desire on the part of the mass media for a return of institutional containment. Here, clusters of feeling becoming briefly ‘unstuck’ from their previous organisations, creating a moment of affective flux.

## Introduction

Much has been written about the history of long-stay psychiatric hospitals in England. From the rebuilding of Bethlem Hospital in 1676 and throughout the next two centuries, the asylum is often presented as the major means by which English society coped with disruptive or uncontrollable mental distress and unmanageable social behaviours. While the 1930 Mental Health Treatment Act replaced the term ‘asylum’ with ‘mental hospital’, the image of institutional care associated with asylums endured powerfully into the 20th century. As numerous histories tell us, large-scale mental institutions remained the primary mode for dealing with distressed or disorderly individuals until the 1950s, even as debates raged about what kinds of treatments or controls should take place behind the asylum walls (Berrios 1991; Porter 2003; Scull 2015). Since its inception, this institutional treatment of the ‘mad’ has had its counterpart in decentralised care taking place in family or smaller private settings (Andrews and Scull 2003; Scull 2006; Smith 2020). Nevertheless, it was these institutional spaces as the most likely destinations for those believed to be insane which loomed large in the public imagination. As Andrew Scull suggests, ‘such mansions of misery rapidly acquired a visibility and cultural salience out of all proportion to their actual numbers […] (T)he image of the madhouse, and the gothic fantasies about what transpired behind its high walls and barred windows, acquired an ever greater hold over the public imagination’ (Scull 2006).

This article investigates the representation of psychiatry and mental illness in the tabloid press, at the moment when this hold finally loosened. Mental hospital discharges began to accelerate significantly from the mid-1950s and the following 40 years saw an increasing political will to close asylums and long-stay psychiatric wards (Scull 1977; Tomlinson and Carrier 1996). In his famous ‘water towers’ speech in 1961, Health Minister Enoch Powell promised a 50% reduction in hospital beds over a 15-year period. In fact, the rate of discharge was so accelerated that by 1971 the Department of Health and Social Security was anticipating ‘*the complete abolition of the mental hospital system* within fifteen to twenty years’ (Scull 1977, 29). This process of deinstitutionalisation of psychiatric patients was driven by financial expediency; an increasing disillusionment with the efficacy and ethics of institutional care; the increasing effectiveness and psychopharmaceuticals; and a series of scandals exposing the mistreatment of patients in long-stay psychiatric institutions (; Hilton 2017; Payne 1999; Taylor 2015, Wallis 2016). As Sarah Payne notes, ‘the principle of caring for people with mental health problems outside of hospital has been welcomed, particularly by user groups and survivor networks’ (Payne 1999, 244).

The deinstitutionalisation of mental hospital patients made its way into statutory law in 1990, in the form of the NHS and Community Care ACT 1990. The Act helped to usher in the final stage of asylum closures by moving the responsibility for the long-term care of mentally ill individuals, as well as those with physical disabilities and learning difficulties, out of the NHS and into the hands of local authorities. The Act redrew the boundary between health and social care in a wide-reaching restructure that continues to impact the health and social care split today. In ‘one of the most far-reaching and significant organisational changes of the period, affecting all health and social services activity’, it restructured health and social care funding around a system of local purchasing (later called commissioning) (Welshman and Walmsley 2006, 84). Jan Walmsley notes,

the 1990 Act is one of the landmark pieces of legislation behind the organisation of community care […] It was in a large part motivated by the need to curb social security payments for residential care. The open-ended, nationally funded and controlled budget for care was replaced by a cash-limited locally administered budget only for those users who were individually assessed as requiring support. Purchasers purchase care on behalf of clients who have been assessed as requiring them (Welshman and Walmsley 2006).

The final psychiatric hospitals and long-term psychiatric wards were thus forced to close in the early 1990s, as accommodating these residents was no longer in the purview of the NHS (The deinstitutionalisation of geriatric psychiatry patients did not follow the same pattern, however, and is often overlooked in accounts of this era; Hilton 2017).

This article examines the reaction to the introduction of ‘community care’ into public and political discourse around the time of the NHS and Community Care ACT 1990 in the tabloid press in order to reveal the consequences not so much of the closure of psychiatric hospitals, as the final, public announcement of their closure: the destruction of the *image* of the asylum and the symbolic function that it had served. I shall continue to use the term asylum rather than mental hospital to describe this symbolic function, as it is a symbol that draws more from the historic legacy of ‘asylums’ and madhouses than from contemporary hospital care. In the late 20th century, asylums served a symbolic function in reinforcing patterns of emotion which had historically been associated with madness and its containment even as they ceased to actually exist. Asylums continued to shape the public image of madness and psychiatry well into the second half of the 20th century. It took this high-profile and polemical piece of legislation and a series of high-profile and violent scandals (Hilton 2017, Wallis 2016) to bring the reality of asylum closures into the eye of the UK public, destabilising a hitherto fixed emotional field in which stereotypes relating to madness, gender and violence interlocked and reinforced one another.

In the UK, tabloid journalism provides a striking and highly affective discursive field for public debates on madness. While tabloids provide a specific and limited prism through which to understand public debates, it is often through their pages that public anxieties and moral panics are reflected, magnified and spread to wider audiences (Cross 2010). Standing on either side of the political spectrum, the *Sun* and *The Daily Mirror* were the most widely read UK newspapers throughout the 1980s and 1990s, both with a daily readership of over three million (United Newspapers 1985). The circulation of *The Daily Mirror* began to decline after 1990 to just under three million readers a day. Nevertheless, it remained the second most popular newspaper until the end of the decade, when its place was taken by the *Daily Mail* (United Newspapers 1985). This article focuses on these publications as vectors of emotion attached to the idea of madness, taking an approach derived affect studies (see further) and from critical psychology and media studies, as put forward by Lisa Blackman and Valerie Walkerdine. As Blackman and Walkerdine suggest, the media is one of many technologies of the self through which individuals and communities come to understand themselves and the world around them:

Human subjectivity is not fixed or precultural, [it is] amenable to techniques that isolate the individual from his or her wider social, cultural and historical context […] The media, as one such process, is therefore once again accorded a role through which we come to develop particular understandings about who we are, and indeed, are allowed to be, at this specific historical and cultural moment (Blackman and Walkerdine 2001, 3-43–4).

Writing about the 1990s, Blackman and Walkerdine argue that ‘both psychology (as a body of knowledge) and the media work[ed] together to provide a way of understanding what is normal behaviour’, through the repetitive circulation of images of ‘desirable’ norms and stereotyped deviance (Blackman and Walkerdine 2001, 4). At a time of radical shifts in the treatment of mental illness, the media emerged importantly as ‘one of the places in which the fictions of the human subject are produced and circulated’ (Blackman and Walkerdine 2001, 6).

Emotions are essential to the ways in which such ‘fictions of the human subject’ are produced in the tabloid press. Following Sara Ahmed’s *The Cultural Politics of Emotion*, I understand tabloids as discursive sites which generate and reinforce emotions, and allow them to circulate between different cultural objects. Emotions are not simply reactions to pleasant or unpleasant stimuli. Rather, they are essential to the ways that notions of sameness and otherness are constructed in society. Ahmed suggests ‘that emotions create the very effect of the surfaces and boundaries that allow us to distinguish an inside and an outside in the first place’ (Ahmed 2004, 10). In other words, emotions are central to the creation of otherness, and the ways in which ‘others’ are fixed within social narratives. When emotions are produced over and over by repeated narratives, they become ‘sticky’, attaching themselves to objects (such objects can be people, places and narrative structures): ‘words for feeling and objects of feeling circulate and generate effects […] they move, stick and slide’ (Ahmed 2004, 14). UK tabloids offer a prime example of the ways in which sticky emotions are generated and circulated. The importance of highly emotional content for tabloid journalism was epitomised in the words of Kelvin Mackenzie, who became the editor of the *Sun* in 1981 and oversaw the paper’s near-stratospheric rise in popularity and some of its most famous and polemical headlines. These include the 1982 ‘GOTCHA’ article covering the sinking of an Argentinian ship in the Falklands war, and the *Sun*’s infamous 1989 coverage of the Hillsborough disaster, which is still responsible for boycotts of the *Sun* in Liverpool. For Mackenzie, the tabloid’s primary aim is an emotional and entertaining one, to ‘shock and amaze on every page’ (Johansson 2007, 20). Tabloids create objects of feeling, through the repetition of stereotypes: the page 3 girl, the football hero, the villainous football manager, the corrupt politician and—as we shall see—the deranged madman and the exciting madwoman. Beyond ‘shock and amaze(ment)’, further feelings get stuck to these objects, including hatred, disgust, fear, triumph and arousal.

Tabloids produce feelings which become stuck to the bodies of ‘mentally ill’ subjects through the repetition of specific and culturally contingent narratives. By focusing on the role of the asylum in these narratives, this article presents the generation of objects of feeling as dependent on the availability of recognisable and stable symbols. The argument follows three stages. First, it reveals the symbolic function that the asylum held in the popular image of psychiatry until 1990 by examining the repeated presence of ‘asylum stories’ in the 1980s tabloid press. The image of the asylum in these stories is a vague one, which blurs the lines between therapeutic and carceral settings to present a consistent and, above all, solid symbol of separation between civilised society and certain forms of disorderly violence and emotion. Asylum stories in tabloids provide an important framework for understanding how mentally ill subjects are transformed into objects of hate and disgust in the popular press, through an essentially melodramatic approach to both madness and gender. These stories also repeat the actual sexual segregations of psychiatric hospitals, through which seemingly ‘unreadable’ behaviours are sorted, understood and contained.

Second, the article reveals the extent to which a constellation of public events, including the 1989 *Caring for People* white paper, the subsequent *NHS and Community Care Act* and the *Inquiry into Homicides and Suicides by Mentally Ill People* (1991), announced the already existent closure of asylums to the media and the public. In doing so, it also removed the asylum as a stable symbol of separation and containment. In this moment, the elimination of the asylum was performed to the public. The Act and its narrative treatment can be considered performative in a Butlerian sense, insofar as it brought together the corporal reality of the closure of psychiatric hospitals (which had been ongoing for 30 years prior and was almost complete at this stage) and the discursive announcement of a new reality: the ‘community care era’ (Butler 1990, 185). This discursive reality, related but not identical to the infrastructural transitions taking place in psychiatric care, was one in which the asylum was announced as absent, conspicuously eliminated from the social world. ‘Community care stories’ come to replace asylum stories in the tabloids following 1990, generating their own distinct objects of emotion.

Third, it argues that by performing the abolition of asylums, the introduction of community care into public discourse appears to have triggered (or contributed to triggering) a dislodging of the norms by which madness and gender had historically been married. In the realm of tabloid journalism, the Act deprived story-tellers of a discriminatory image through which to filter disruptive ‘psychiatric’ behaviours and, in doing so, opened up a gap in which different kinds of psychiatric stories began to be reported. These stories rest less heavily on gendered binaries while also demonstrating a near-frantic desire on the part of the mass media for a return of institutional containment. Community care stories disrupt the expected emotional terrain that had been attached to the symbol of the asylum, and clusters of feeling become ‘unstuck’ from some gendered and institutional stereotypes.

Due to its focus on sensational public discourse, the voices of those directly impacted by the changes in treatment in the period are not present in this article. The aim here was not to sideline these voices but to demonstrate how they were largely removed from the popular press in an intensely objectifying and emotional tabloid landscape. Beyond the tabloid newspapers, community care was a widespread object of concern in the 1990s. Patient groups and practitioners expressed concern that deinstitutionalisation led to homelessness as well as a personal feeling of dislocation, and not understanding one’s role in society (Barham 1992). Other cultural media also wrestled with the question of how to represent psychiatric subjects in this period. On television for example, Channel 4’s 1999 drama *Psychos*, opened with an episode structured around the community care murder plot, focusing on the disastrous decision to discharge a violent psychiatric patient. *Coronation Street* ran a long story throughout the 1990s of a vicious woman murderer with ‘erotomania’. In contrast, the 1994 BBC2 series *Takin’ over the Asylum* attempted to challenge these stereotypes by telling the story of a group of young people setting up a radio station in a mental hospital (Franceschild 2008). Here the narratives of entrance, exit or residence in long-term psychiatric care continues to structure representations of mental illness. The same might be said of theatrical productions in the 1990s. Theatre returned to the theme of madness with renewed interest in this period, with a new generation of writers such as Joe Penhall, Andrew Nielson, Sarah Daniels and Sarah Kane staging ‘community care plays’ (Harpin 2018). These plays focus on the so-called revolving door problem of community psychiatric care, with protagonists constantly entering, leaving and re-entering psychiatric locations.

## Walls, cages and column inches

The image of the mental institution, in the form of the asylum, mental hospital or secure psychiatric ward, looms large in tabloid reporting of mental illness in the 1980s. As described earlier, long-stay psychiatric hospitals had been in decline for almost three decades by 1980. Inpatient psychiatric services had been in steady decline since the 1950s, with more and more patients treated through outpatient care or brief stays in acute wards (Tomlinson and Carrier 1996). In spite of this, an examination of mentions of psychiatry and madness in two major tabloids of the 1980s reveals that a hybrid version of the asylum endured in the public imagination.

In much popular discourse in the 1980s, the term psychiatric was associated with high-security psychiatric facilities, typified by the frequently mentioned and ominous Broadmoor. In the *Daily Mirror* and the *Sun* in the 1980s, psychiatric wards are presented as performing an essential function inadequately, of walling in and separating perpetrators of perverse violence from the general population. Using a language of locking up, caging and walls, these articles contain clear echoes of 19th-century gothic representations of asylums (Porter 1987). They present psychiatric hospitals stereotypically as sites of horror and suggest that any mixing between psychiatric and social spaces is deeply threatening.

Discussion of mental illness and psychiatry is shaped in the 1980s tabloid press by these asylum stories, which we can understand as generating powerful *objects of feeling* for the reader. In the opening to her study *The Cultural Politics of Emotion,* Sara Ahmed introduces the idea of objects of feeling as generated through repeated and familiar narratives. Such narratives create feelings through the demarcation of an inside and outside, inviting ‘you’ into a ‘we’, and directing this collective identity against an other who is rendered up as an object to be feared, hated, loved etc. It is the combination of repetition and invitation in these narratives which, for Ahmed, causes them to ‘press’ onto the minds and bodies of their readers and listeners, moulding feelings to become attached to particular objects (Ahmed 2004, 6). Repetition has been essential to the generation of predictable affects in popular publications since the rise of 18th-century periodicals (Dillane 2016). As Greg Philo and the Glasgow Media Group have demonstrated, these stories become so powerful and insistent that they can actually override beliefs based on personal experience (Philo 1996). Such narratives gain power through their circulation, especially in the tabloid context. Tabloid papers are highly circulated, both in the sense that they are popular, and in the sense that they are essentially social texts. As Sofia Johansson’s study of tabloid reading habits demonstrates, these papers themselves are objects of feeling and circulation. The tabloid paper is thrown down in disgust, passed around the canteen, offered to fellow commuters, and discussed at length in domestic and work places. Consumers buy tabloids, according to Johansson, in order to be entertained and to ‘have something to talk about’ (Johansson 2007, 149). This sociality of the tabloid reinforces the invitations to collective identity that the stories themselves contain.

The asylum stories in tabloids in the 1980s create objects of feeling by a repeated use of the image of the asylum or psychiatric ward as a container for deviant persons. The image of psychiatric sites as inadequately performing an essential act of separation is summarised clearly in an article in the *Daily Mirror* from 1981, entitled ‘Boy is Caged with Madmen’. (Boy 1980) This article reports the presence of a 12-year-old boy in Moss Side Special Hospital, which had been converted to a high-security psychiatric hospital following the 1959 *Mental Health Act*. The article reports the case of a ‘boy of twelve (who) is being held in a top security hospital for the criminally insane. He is among six youths, all under 17, at Moss Side Special Hospital, near Liverpool. Similar hospitals house murderers and psychopaths (‘Boy’ 1980, 5). The article expresses anxiety at the contagious mixing of incompatible categories and, in doing so, produces the hospital and its inmates as objects of horror and disgust. A boy of 12, it implies, cannot be in the same category as the ‘murderers and psychopaths’ that occupy ‘similar hospitals’ (Boy 1980). The caging of such people is not being disputed. Rather it is presented as outrageous that a child, usually associated with innocence and vulnerability in much tabloid writing of this decade, should be sharing a space with perpetrators of monstrous violence (an assumption with would be profoundly shaken by the Jamie Bulger case in 1993).

The description of the special hospital in this article, though brief, reproduces ideas that had been associated with asylums since their inception, and which were reinforced in much mid-20th-century writing about the purposes mental institutions used to serve (see Tomes 1995). Writing about the creation of 18th-century asylums in his influential antipsychiatric book *Madness and Civilisation,* Michel Foucault identified the modern asylum as a site in which violence and unreason are silenced, contained and displayed:

The ideal was an asylum which, while preserving its essential functions, would be so organized that the evil could vegetate there without ever spreading; an asylum where unreason would be entirely contained and offered as spectacle, without threatening the spectators; where it would have all the powers of example and none of the risks of contagion. In short, an asylum restored to its truth as a cage (Foucault 1988, 207).

‘Boy Is Caged with Madmen’ evokes this image of the asylum as cage and highlights the exclusionary function which the the secure psychiatric facility is assumed to perform in the popular press of this period. The article does not refer to the treatment of the youths in Moss Side, nor does it consider the nature of Moss Side as a specific site (Moss Side would find itself at the centre of an abuse scandal less than a decade later). Special hospitals are grouped together as the natural locations of unnatural types, the murderers and psychopaths. Moss Side is attacked for allowing such individuals to share space with those who do not belong to their category and facilitating a dangerous mixing between perversity and vulnerability and innocence.

It is through this mixing that the special hospital becomes an object of horror: epitomised in the very headline: Boy Is Caged with Madmen. In her writing on abjection, Julia Kristeva defines ejected bodily material as causing horror and repulsion because it threatens a discursive breakdown between subject and object:

It is thus not lack of cleanliness or health that causes abjection but what disturbs identity, system, order. What does not respect borders, positions, rules. The in-between, the ambiguous, the composite (Kristeva 1982, 4).

Horror is generated for Kristeva through borderlessness, typified by the revulsion that we feel when viewing bodily fluids, parts or corpses. However, a narrative object can equally generate feelings of horror and disgust. We can readily imagine reading a headline such as Boy Is Caged with Madmen and throwing down the paper in disgust, or exclaiming ‘that’s disgusting!’. Ahmed builds on Kristeva’s formulation by suggesting that disgust simultaneously reveals and threatens the border between inside and outside precisely in order to reinforce this binary:

Perhaps the ambiguity relates to the necessity of the designation of that which is threatening: borders need to be threatened in order to be maintained, or even to appear *as* borders, and part of the process of ‘maintenance-through-transgression’ is the appearance of border objects (Ahmed 2004, 87).

The headline and ‘asylum story’ can become such a border object. It reminds the reader that there is a space in which those other madmen are usually contained, even while it presents the border of this site as violated through the presence of a child. As such, it repeats other narratives of horror, for example, those of popular asylum horror movies, in which the narrative’s affective force is located in the conviction that the protagonist is locked up *in the wrong place*. The trope of waking up in an asylum or ‘nightmare factory’ and not knowing how one got there is the starting point of classic asylum horror movies such as *The Snake Pit* (1948), a genre which emerged in the context of the beginnings of deinstitutionalisation of psychiatric patients in the USA (Erb 2006; Rondinone 2019). More contemporary versions of this trope include the 2003 psychiatric horror *Gothika*, in which Halle Berry plays a psychiatrist who inexplicably wakes up as a patient in her own secure psychiatric ward. In such cases, horror is generated through a frightening mixing of sane subjects in insane places.

While there has been much debate about the applicability of Foucault’s analysis to UK psychiatric history, the idea of the asylum as cage is a powerful and relevant one here (Scull 1990). Recent histories of psychiatry have challenged the idea that the mad were exclusively, or even predominantly, treated in long-stay institutions in the 18th and 19th centuries (Smith 2020; Scull 2006; Tomes 1995). Topp, Moran, and Andrews 2007 suggest, for example, that an excessive focus on asylums in both historical and popular accounts of psychiatric history has ‘not only obscured the complexity of asylums, but also overshadowed the many other types of built space in which madness has existed’ leading to a ‘strategic oversimplification […] in historical accounts of spaces ‘reserved for madness’ (2007, 1). The separation of the ‘mad; from civil life has always taken diverse forms, including confinement in private residences, therapeutic communities and financial disempowerment. In part due to the influence of Foucault and other anti-psychiary writers such as Irving Goffman, this complex history was obscured in the mid twentieth century, by the powerful and singular image of the asylum as an ‘undifferentiated black hole for society’s unwanted’ (Topp, Moran, and Andrews 2007, 2). It is this image of asylums that is emotively deployed in the 1980s tabloids.

Tabloid newspapers of the 1980s demand that psychiatric services act like a symbolic asylum, ensuring the separation of the ‘normal’ and the mad. Asylum and ‘psychiatry’ are elided in these stories, with the term psychiatry acting metonymically as a stand-in for a number of different sites, including special hospitals, long-stay psychiatric wards and acute psychiatric settings. These settings are presented as though their primary role is one of confinement, even where this is not in fact the case. Articles mentioning psychiatry in the *Daily Mirror* from 1980 to 1990 can be broadly broken down into three categories: (1) those about men being sent to psychiatric hospitals or for psychiatric testing after committing a violent or sexual crime (62 articles); (2) those about women who have either been committed to psychiatric hospital/received psychiatric tests having been victim to a violent or sexual crime or to domestic abuse, or who have been abused while in a psychiatric facility (or both) (45 articles—there were 8 additional articles that referred to abused children in a similar light); and (3) those reporting on systemic problems in the NHS, including underfunding, neglect and mistreatment in psychiatric hospitals and wards, and psychiatric treatment in prisons (44 articles). (This is based on a text-based search of articles mentioning psychiatry or psychiatric in the *Daily Mirror* and *Sunday Mirror* from 1980−1990.) In addition to this were articles that either reported the breakdowns (6), eating disorders (3), or suicides (9) of famous or infamous figures. The remaining articles either reported psychiatry incidentally (10) or related to the following: psychiatric treatment following road accidents; alcohol or drug abuse; nervous breakdowns unrelated to abuse or violence (3); and general medicine (2), women’s health (4), world health (2), LGBT rights (1) and one human interest story about a rat. There are also two articles which report women perpetrating violent crimes and being sent for psychiatric tests, which do not frame the women as victims, unlike those included in category 2. Clusters of emotion become attached to each of these types of narrative, generating different but related objects of feeling.

The term psychiatry becomes a means of sorting stories of violence into gendered stereotypes in these articles and then packing them neatly away behind the walls of a mental hospital. While the archive does contain some positive or neutral stories (eg, a celebrity discussing her recovery from an eating disorder), it is the stories representing gendered forms of violence which are most frequently repeated. This creates and reinforces an emotional pattern which is pressed again and again on the reader. Reading the *Daily Mirror* articles together, one gathers a cumulative picture of the psychiatric ward as a site to which male perpetrators of outrageous, sensational or seemingly perverted acts of violence are sent. In other words, psychiatry almost always means forensic psychiatry, and its male subjects are described for the reader as objects of hatred and rage.

One article which made the front-page headline in 1983 clearly demonstrated the perception of psychiatric treatment as a way out for men with dangerously perverted minds. The headline reads: ‘Revenge Attack Father Is Jailed’, with the byline ‘he beat up the sex fiend who assaulted his daughter’(Revenge 1983, 1). The article reports the jailing of a ‘father’ for ‘beating up a man who had sexually assaulted his eight year-old daughter’ (Revenge 1983). The man is said to have been ‘goaded’ into this act of vigilantism ‘because he felt the law had not punished [his daughter’s attacker]’, after the latter was sentenced to receive psychiatric treatment rather than a prison sentence (Revenge 1983). The article constructs psychiatric treatment as an outrageously light sentence compared with the weight of the crime, implicitly lending its approval to the fathers’ vigilantism. Having stated that the child’s attacker went ‘unpunished’, the article goes on to provide a detailed description of the wounds the father’s attack inflicted on him: ‘He kicked in the door of the house and attacked him, giving him a fractured jaw and nose, two black eyes and severe cuts and bruises. The man was in hospital for two weeks’ (Revenge 1983). Providing dramatic details of the staging of the ‘revenge attack’, the article solicits identification with the father and offers the wounded sexual predator’s body as an object of hate for the reader, as a kind of symbolic reassurance that at least one form of justice has been done.

This article clearly lays out a distinction between two forms of violence which is found repeatedly in tabloid articles throughout the 1980s. On the one hand, the violence of the father is presented as understandable. The reader is invited to relate to the man who commits violence to avenge his disabled, 8-year-old daughter, and the article ends with a comment from his wife asking: ‘what would any other father have done?’(Revenge 1983). In other words, the father’s act of violence is presented as normative and even relatable, and follows a predictable pattern of emotion and masculinity which the reader can follow. The violence of the other man, however, is of a different sort. We are told nothing about this man other than the fact that he is a ‘sex fiend’ and ‘a 47-year-old paedophile’ and that he has received psychiatric treatment. This form of perverse, illegitimate violence is presented as *the* male mental pathology in popular references to mental illness in this period, its destination always some form of ‘psychiatric treatment’. As Cross (2010) notes, this destination is deeply criticised in tabloids in this period, whose attempts to ‘assert […] moral governances over murderers [and perverts] is thwarted when [they] end up not in prison but in *hospital*’ (117 original emphasis).

By reading this subgenre of asylum story as creating objects of hate, we can also see its role in legitimising a normative male violence. As Ahmed notes, ‘Hate is always hatred of something or somebody’ (2004, 49). What’s more, hatred (unlike horror) creates and sustains a stable opposition between the hating and the hated. Indeed, much psychoanalytical writing on hatred emphasises the extent to which the hated object becomes essential to the hating subject: ‘What is at stake in the intensity of hate as a negative attachment to others is how hate creates the ‘I’ and the ‘we’ as utterable simultaneously in a moment of alignment’ (Ahmed 2004, 51). (We might also consider the central role that hatred plays in the Melanie Klein’s model of projective identification, in which the generation of a hated object through the projection of the hated parts of the self into an other is an essential step in subjectification (Klein 1990, 181.) In this story, the reader is invited into a moment of hatred for the body of the paedophile, through the wife’s invitation to identify with his attacker: ‘What would any other father have done?’ The reader is interpolated into a ‘we’ that understands normative male violence through a lens of paternal love, which is sustained through the hatred of an alternative violence which is insane and perverse.

On the other side of the gender divide, tabloid representations of mentally ill women in this decade invariably depict younger women according to a sexualised victim narrative. According to this narrative, women with psychiatric problems are caught in a double bind. Either they are institutionalised as a consequence of sexual abuse, or they become victims of physical or sexual abuse while they are in psychiatric institutions. One major story relating to the psychiatric detainment of women, for example, broke in November 1980 when newspapers and an ITV documentary reported the plight of a young woman, ‘Christine’, who had been detained for over 4 years on an ‘indeterminate life sentence’ in a psychiatric unit due to aggressive, though non-criminal, behaviour. The article covering this story in the *Daily Mirror* takes up the upper half of a double page spread and with a large accompanying photograph. The article paints Christine, dubbed ‘The Girl That Life Forgot’ in the headline, as a victim of the care system (Girl 1980, 12). Her physical self-harm is graphically described and the blame pointed squarely at the authorities detaining her.

As well as revealing and attempting to generate a deep distrust in the psychiatric and social care systems, the article also sexualises the young woman whose plight it claims to reveal. As such, it introduces a third kind of emotional object related to psychiatry, separate from both the objects of hatred and horror we have seen so far. While Christine’s behaviour is described in a quotation from a MIND representative as ‘aggressive’, the journalist penning the article describes her ‘tearaway exploits’, generating the image of rebellious, exciting adolescence (Girl 1980). The photograph accompanying the article is of a wide-eyed and attractive Amanda York, half concealed behind bars, the actress in the dramatisation which would accompany the documentary about Christine. The article is thus careful to present Christine in terms of a traditionally misogynistic and accessible version of femininity, for all the transgressive behaviour which has located her in the realm of the psychiatric in the first place.

The presentation of female sexuality in this asylum story clearly fits the gendered style of tabloid reporting more broadly. Johansson identifies the regulation of female sexuality as a key feature of the ways in which women are represented in the *Sun* and the *Daily Mirror*. In her thorough study of the Entertainment sections of both papers (sport, gossip pages, page 3 and advice columns), Johansson explores the possibility that tabloids might be presenting female sexuality as powerful or liberated. However, a closer look at each instance reveals a clear characterisation of women as wildly sexual, to be contained and displayed by the tabloid itself. This takes place in different ways in the different sections. On page 3, the young woman’s sheer availability as an object of consumption for the male reader contains any supposed ‘confidence’ she may be displaying (Johansson’s reader research fascinatingly shows that despite the *Sun*’s claims, its regular female readers have viscerally negative reactions to page 3) (Johansson 2007, 184). Scantily clad or sexually active female celebrities are routinely shamed in both the *Sun* and the *Daily Mirror* gossip columns. In the meantime, advice columns routinely publish letters from men and women addressing the ‘problem’ of female sexual appetite, under headlines such as ‘My Gorgeous Girl Wears Me Out with Her Sex Demands’ and ‘Husband Refuses My Amorous Attention Once Again’. Such problems are submitted to agony aunts, ‘motherly older women’ who advise strategies for such women to curtail their sexual appetites (Johansson 2007, 110). Given the dominance of this model of female sexuality across both publications, it is unsurprising that women in psychiatric stories are also presented through this prism of transgressive-yet-available sexuality.

The combination of victimhood, passivity and provocativeness in female madness has a long cultural history. In the 1960s and 1970s, second-wave feminist historians of psychiatry argued powerfully that the regulation of female behaviour and sexuality has been historically embedded in psychiatry since its inception. Feminists such as Chesler 1972, Russell 1995 and Showalter 1987 presented the history of psychiatry as a violently norming social and cultural force which pathologised female desires, emotions and freedom. More recent studies have attempted to provide a firmer understanding of the mechanisms by which psychiatry might operate a (sometimes unwitting) medical misogyny. Busfield 1996 carefully rebuts the assumption in these histories that psychiatry has pathologised femininity per se, arguing instead that ‘there is an indirect relationship between gender and the official constructions of mental disorder […] because, although formally described in universal terms, the categorisation of specific disorders refers to mental life and behaviour that is to a greater or lesser extent gendered’. Clearer instances of psychiatric sexism are found in Joe Sym’s research into the continued use of standards of ‘sexual respectability’ in prison psychiatry and in the sexist implications of the borderline personality disorder diagnosis which have been highlighted by researchers and patient groups (Appignanesi 2011; Sim 2005; WISH (Women in Special Hospitals) 2005). Underlying these concerns is the idea that female sexuality is both available for diagnostic consideration and in need of regulation.

Whereas the asylum stories about men and psychiatric incarceration in the 1980s generate objects of horror and hatred, those about women and institutionalisation are written as transparent and titillating to the (presumably male) *Daily Mirror* reader. Articles about the abuse of women and adolescents in the care system are even more explicit. The subheading to an article relating to the forced medication of a female teenager in ‘a unit for disruptive children’, ‘Problem Girl Drugged with Liquid Cosh’ in 1987 reads: ‘A girl of 14 was sedated with the powerful tranquiliser “liquid cosh” to curb her sexy antics’ (‘(Problem 1987, 8). The article plays a double game of quoting a MIND representative condemning the treatment on the one hand, but giving the final quotation to the young person’s care worker describing ‘the girl whose sexy behaviour disrupted work’: ‘“She moved her lips provocatively”, he said. “She stirred up the older boys by the way she used her body”’ (ibid.). Another article relates how a psychiatric ward has had to use smoke bombs to flush out the ‘boyfriends’ of female patients to stop them having sex (Hospital 1984, 4). The trend in presenting female psychiatric patients therefore treads a line between presenting them as victims and in this presentation sexualising and victim-blaming them. We therefore might describe these stories as objects of arousal masquerading as objects of outrage. Read together, the articles build up a fantasy of the hypersexual, mentally ill young woman held at the mercy of a system which punishes her for her apparently unstoppable sexual behaviour.

The supposedly rampant nature of this sexual behaviour echoes the animality presented in the articles about male psychiatric subjects. Female provocation forms a parallel with perverse male violence because it too is both outside of responsibility (outside of the subject’s control, pathological) and simultaneously merits blame and punishment. *Daily Mirror* articles of the 1980s maintain a narrative of mental pathology which is bound up with the idea of a site in which such unstoppable passions might be contained—the asylum. As I have argued, these articles bring together special hospitals, secure psychiatric wards, care homes and inpatient psychiatric wards to represent a single symbolic site. This site does normative gender work: punishing hypersexual women and containing (but failing to adequately punish) illegitimately violent men. ‘The psychiatric’ here is indeed a Foucauldian cage. It is a site of containment for forms of violence that can only be understood through the psychiatric prism. It anchors these forms of violence to a (medical) discourse which renders them legible while also enacting physical/topographical containment on its perpetrators. The tabloid itself becomes the gaps in the cage’s bars, as it is lays out the scandals of the psychiatric institution and the crimes of its patients for its readership as sensational spectacle.

The discursive ‘caging’ of the psychiatric subject is thus performed on both male and female subjects. The female psychiatric subject is presented as unwittingly, uncontrollably (animalistically) sexual, and her story is served up as spectacle for the male reader. With the subject safely held in by both the walls of her ward/asylum and the tabloid’s column inches, the reader is invited to enjoy her story, even as he is allowed to preserve the position of moral righteousness and outrage, given that the articles are ostensibly critical of her victim position. Like 18th-century visitors to Bethlem asylum, the 1980s tabloid reader is invited to gawk and perform outrage in equal measure. Scull’s summary of the actions of Bethlem’s visitors has echoes here:

If more and more of the hoi polloi came to gawk and laugh, to view inmates in a peculiarly human sort of zoo, those who thought themselves their social superiors and moral betters began to parade their own sense of sorrow, mortification and disgust, maximising the distance between polite and popular culture, and in doing so making manifest their own more refined sensibilities (Scull 2006, 17).

The point here is not simply to draw parallels between 18th-century and 19th-century asylum visitors and tabloid readers. Rather, it is to suggest that reporters as late as the 1980s continued to draw on and write within tropes of confinement, perversity and spectatorship which had been embedded in English cultural attitudes towards mental illness for centuries. In Ahmed’s terms, the image of the asylum or psychiatric institution becomes ‘stuck’ to a set of gendered emotional clusters, generating objects of feeling which perpetuate already deeply entrenched stereotypes.

## Community care: shifting the emotional terrain

Evidence from archives of the *Daily Mirror* and the *Sun* newspapers suggests that the link between historical asylum narratives and popular representations of psychiatric spaces took a dramatic turnaround 1990. At this moment, a number of high-profile events introduced the phrase 'community care' sharply into the public eye: the 1989 White Paper ‘Caring for People: Community Care in the Next Decade and Beyond’ the subsequent passing of the NHS and Community Care ACT (1990) and the *Confidential Inquiry into Homicides and Suicides of Mentally Ill People* which was started in 1991. Tabloid reporting of psychiatric stories undergoes an affective shift in this period, introducing a moment of narrative flux.

The NHS and Community Care ACT 1990 (Community Care Act) marked a turning point in the treatment and perception of psychiatric patients and mental health service users in England. The Act had three major consequences for UK mental healthcare. On an economic level, it enacted a shift in the way the NHS was to be conceived of and managed. The Act created the NHS internal market and legislated for the release of resources from newly formed NHS Trusts to Local Authorities for the delivery of social care in the community. At the level of service–provision, it consolidated the process of deinstitutionalisation of physical and learning disabled, mentally ill and chronically ill patients which had been steadily taking place for several decades. On a cultural level, the Act and the changes it suggested provoked a crisis in the perception of mentally ill patients in the general public, exemplified by a series of ‘community care murders’ which occupied the press throughout the decade. These changes came together to produce a radical shift in the cultural discourse surrounding mental illness, as they brought about a decisive split between the asylum and the contemporary psychiatric subject in the public imagination.

In both economic and social terms, the Community Care Act paved the way for the partial privatisation and neoliberalisation of psychiatric care, and the NHS more widely. As Walmsley and Welshman note in their study on community care and adults with learning disabilities, community care services already existed throughout the 20th century as adjuncts to institutional care. The real shift typified by the Act, they argue, was the conversion of such services into adjunct support to family care (Welshman and Walmsley 2006, 9). In both mental healthcare and care for those with learning disabilities, the Act caused a shift from ‘monolithic provision of services by the NHS or social services’ to a purchaser/provider model, constituting ‘one of the most far-reaching and significant organisational changes in the period, affecting all health and social service activity’ (Welshman and Walmsley 2006, 84). The Community Care Act thus shifted the locus of care for those with mental illness, physical disabilities and learning disabilities away from the medical sphere and into private spaces. In this new model, ‘the open ended, nationally funded and controlled Department for Social Security budget for care was replaced by a cash-limited, locally administered budget only for those users who were individually assessed as requiring support’ (Welshman and Walmsley 2006). As Walmsley and Welshman note, ‘the rhetoric of choice (was) extensively deployed to justify this marketisation’, while in fact the choices offered to service users, especially those with severe mental illness or learning disabilities, were almost always made by the care provider and not the individual (Welshman and Walmsley 2006).

This dislocation of mental healthcare from the site of the asylum provoked immediate outrage in the popular press, revealing a pervasive anxiety as to the failure of public institutions to control ‘dangerous’ psychiatric patients. Three particularly high-profile murders by former psychiatric patients dominated the press during the early 1990s, and the Community Care Act was framed as directly leading to these tragedies (although in fact it was loopholes in The Mental Health Act (1984) which in part allowed them to happen). The media storms surroundoing these murders were prolonged and magnified by the publication in 1994 of the *Confidential Inquiry into Homicides and Suicides of Mentally Ill People*. As Philo (1996) has revealed, the results of the Inquiry were widely misreported, leading to an inflated notion of how likely these attacks were. Following the murder of a young girl by psychiatric outpatient Carol Ann Barratt in 1991, Fred Graver and Jonathan Zito were both killed in 1992 by patients who had similarly recently received psychiatric care. Of these, the murder of Jonathen Zito by Christopher Clunis gained a very high profile. In her history of the political changes affecting the NHS in the 1990s, Anne Richardson suggests that it is difficult to underestimate the impact that this murder had on public and political opinion, and she notes that ‘all (government ministers) were very affected by the events surrounding the death of Jonathan Zito’ (Richardson 2015, 85).

The reporting of Zito’s and of Graver’s deaths in the *Sun* and the *Daily Mirror* both emphasise the role of community care in the murders. Articles from the early 1990s frame community care policy as freeing dangerous individuals who ‘should not have been allowed out on the street’(Hay 1993, 8). By the end of the decade, the *Sun* was carrying out a concerted campaign to end community care murders by putting mentally ill individuals ‘back’ into long-term institutions—demanding that ‘psychos […] be taken off the streets and caged for life’ (Gilfeather 1999, 14). The phrase community care is invariably linked to violence in articles in the *Sun* throughout the 1990s, often appearing under scare-mongering headlines like ‘Sick Killers Toll of Misery’ (Reynold 1999, 15), ‘Two Women Die in Stab Frenzy’ (O’Reilly 1997, 1) and ‘Scandal of Psycho Freed to Kill Hero Cop’ (Sullivan 1998, 4). The Community Care Act was consistently understood as a dangerous piece of legislation in much popular press and television coverage in this period.

These community care stories replace the earlier asylum stories and offer a palpable shift in the emotional terrain of tabloid reporting on mental illness. As I have argued, the symbol of the asylum contained stories of illegitimate violence until 1990, enabling a narrative structure which offered mad subjects up to the reader as *distant* objects of hatred, horror or arousal. After the introduction of the Act, these stories changed shape, becoming concerned with the proximity of mad subjects to normal life and generating them as objects of fear. This is a subtle but important shift. Whereas asylum stories created and reinforced boundaries between the ‘sick’ and the normal, headlines such as ‘Scandal of Psycho Freed to Kill Hero Cop’ emphasise the absence of such a boundary, and the proximity of danger. Part of this danger is that mad subjects are no longer easily recognisable. They become objects of fear precisely because they could well be ‘walking the street’ unnoticed. Ahmed notes:

The more we don’t know who or what we fear, *the more the world becomes fearsome*. In other words, it is the structural possibility that the object of fear might pass us by which makes everything potentially fearsome. This is an important dynamic of the spatial politics of fear: the loss of the object of fear renders the world itself a space of potential danger, a space that is anticipated as pain of injury on the surface of the body that fears. (Ahmed 2004, 69 original emphasis)

Community care stories highlight and create the lack of boundary between psychiatric and public space, generating an impression of lurking danger. Community care becomes a narrative flag in these stories, to which feelings of fear get stuck. Tabloids in particular presented community care as the psychiatric establishment washing their hands of their social and professional responsibility to regulate and contain violence.

Tabloids reporting on community care in the 1990s drew on polemical stereotypes to emphasise the danger of former patients mixing with the general population. While all community care murders are presented as monstrous, it is Zito’s death that is most often reported in accounts of the period as having had a wide public and political influence. This is partly due to the actions of Zito’s wife, who launched a high-profile campaign to amend the Mental Health Act following her husband’s death. At the same time, there were certainly racial and gendered politics to the reporting of Zito’s death. Throughout the 1990s, Clunis was singled out as the most important example of ‘community care gone wrong’. In line with the historical and continuing presence of racial prejudice in the psychiatric system in the UK and in public perceptions of mental illness, the narrative of a black psychiatric patient murdering a white, middle-class, ‘sane’ man exercised a particular hold on the public imagination (Fernando 2002; Fernando 2010). As Blackman (2001) suggests:

Media reports at this time, in line with the repeated way in which mental health signifies with the broadsheets and tabloid press, brought into play an associated set of signifiers which constitute ‘mental illness’ as sick; dangerous; a risk; a timebomb waiting to go off. […] These signifiers were hung around the image of Clunis as a large black man who had killed a young, white, married man (2001, 4).

Such a narrative might be seen by the tabloid reader to represent a shocking reversal of historical power structures, by which white sanity has (violently) regulated supposedly violent black ‘madness’ for centuries. In this context, the violence that was reportedly unleashed by community care is reported not as a series of unfortunate misdiagnoses but a sudden loss of structures of hierarchy and control.

The repeated signposting of crimes as community care murders in the tabloids following 1990 points to an emotional and symbolic importance which the Act and associated community care policies took on, which was disproportionate with its actual effects. From within mental health services, both patients and practitioners felt that community care had taken on a mythic quality. Practitioners and patients taking part in an oral history project agreed that community care policy became an important symbolic force even before it was adequately implemented:

in this somewhat chaotic climate they themselves, as front-line workers, were significant in shaping, for better or worse, the public policies they were supposedly implementing, when community care was ‘a kind of shared myth’ without clear definition (Turner et al. 2015, 9).

Turner *et al*, have revealed the extent to which the Clunis case shaped the memories of medical practitioners of this period. In this period of rapid change, it triggered a sudden backlash against community care services by policy makers. From the perspectives of the practitioners and patients interviewed by Turner’s team, these scandals caused a U-turn in policy makers’ attitudes leading to a diversion of resources back to practices of confinement in high-dependency and medium secure units.

While this represents the interpretation of those closest to the changes themselves, the reporting in this period shows a divergent public attitude. As argued previously, the image of psychiatry as confinement still loomed large in popular media until the late 1980s, with the gradual move towards community services barely mentioned in the popular press. The term community care emerged suddenly into these discourses in the early 1990s in reports of precisely these kinds of scandals. In this context, community care represented nothing short of an abdication of carceral responsibility, the pulling down of walls that were previously imagined as separating dangerous individuals from the public. Scandals such as the Clunis affair reinforced the symbolic function of asylum in the popular press, even as it shockingly announced the absence of its walls.

## New female maladies? The new place and the person of insanity

The dangerous mixing between madness and society which the tabloids associated with community care seems to have had an additional, surprising consequence. As discussed earlier, tabloid reporting on psychiatry before the Act had largely followed highly gendered stereotypes. The asylum walls, as imagined in these reports, uphold two functions: separating supposedly ‘perverse’ behaviours from normal society, and separating male and female versions of perversity from each other. In both cases, we can be reminded about the role of walls in fantasies of unity and sameness. As Wendy Brown notes in her study of border walls and sovereignty, insistence on wall-building emerges at the moment in which a border is already porous and embedded in inevitable systems of movement and exchange. National border walls, for example, do not materially reduce the smuggling of people or drugs across borders, but they continue to be built. Rather, Brown suggests the building of border walls insists on a fantasy of sameness within the border and otherness beyond it, and performs a symbolic separation of the two: ‘national-state walling responds in part to psychic fantasies, anxieties and does so by generating visual effects and a national imaginary apart from what walls purport to “do”’ (Brown 2010, 109). A similar set of fantasies surrounds the image of asylum walls. As a walled-in space, the symbol of the asylum evokes a fantasy of sameness among those on either side of the wall. Those held in the psychiatric system are reduced to being examples of madness conforming to a few simple stereotypes. Those outside the walls are imagined as occupying a flattened-out normativity, which is diverged from at one’s peril. As Ahmed notes, real and imaginary walls both enforce norms, as technologies ‘that stop us being affected by some bodies: those that might get in the way of how we occupy space’ (Ahmed 2017, 145).

In the tabloid press, the apparent disappearance of asylum walls signalled by the Community Care Act conveys a sense of symbolic and narrative collapse. This sense of an upheaval of racial hierarchies in the reporting of the Clunis story is accompanied by a concurrent anxiety about the relationship between madness and gender. The releasing of female psychiatric patients into the community coincides with a widening of the kinds of stories regarding women patients which appear in the popular press. After 1990, in the *Daily Mirror* archive, the newspaper becomes more frequently concerned with stories of female psychiatric violence. While articles between 1990 and 1995 continue to associate psychiatry with male violence (29 articles), only seven articles in this period follow the narrative of the previous decade with regard to psychiatry and women as victims which is described in the ‘Walls, cages and column inches’ section one above. Twice as many articles in relation to psychiatry report stories of women committing extreme acts of violence and being placed under psychiatric care. The change in reporting attitude before and after the Act goes beyond the preferences of individual editors. The *Daily Mirror* had two editors in the decade preceding the Act, and four editors in the following 5 years. Despite this high editorial turnover, the narratives surrounding psychiatry in these periods are relatively consistent, with 1990 being a very noticeable transitional point in which the gender narrative changes. If, as I have been arguing, the repetition of such narratives generates emotional frameworks for understanding other mad bodies then we might identify this period as on of emotional and narrative flux. These stories display a new ambivalence towards women receiving psychiatric care, as articles veer between framing violent women as victims, and as perpetrators of monstrosity. The reporting of these acts betrays anxiety over the care in the community policy and uncertainty as to how and where to situate perpetrators of these killings.

Reading these articles cumulatively, one receives the impression that new, female forms of violence are suddenly emerging from psychiatric spaces which cannot be contained by the narrative strategies of the *Daily Mirror* reporter. This can be seen clearly in the reporting of the case of Caroll Ann Barratt, who murdered a young girl in a shopping centre in 1991, 2 days after being discharged from a psychiatric ward. On the one hand, the front-page headline of this story clearly frames Barratt as a hateful, genderless monster: ‘Doc Freed Psycho to Kill’ (Oldfield and Hughes 1993, 1). The lead article continues in this narrative vein, referring to Barratt as a ‘crazed psychopath (who) stabbed a little girl’ and laying the responsibility for the child’s death at the feet of the doctor who discharged her (Oldfield and Hughes 1993). Nevertheless, the commentary articles that follow the leader attempt other forms of characterisation. One focuses on Barratt’s life as a psychiatric patient, noting that she ‘was admitted to mental hospital 20 times in 10 years’ and that ‘Barratt was the victim of a serious sexual assault at the age of 13. And that triggered her mental problems’ (Oldfield and Hughes 1993, 5). This article therefore moves Barratt from the ‘crazed psychopath’ to a ‘deranged’, victimised young woman.

The coverage of the Barratt story appears as a collage of narrative frameworks, all trying to account for the same act. The layout follows the tabloid style of ‘earthquake news’, in which a particularly scandalous story is reported over several pages in different features in order to generate maximum impact. A third article on the same page, entitled ‘She Killed a Little Angel’, attempts to understand Barratt’s motivations by painting her as the victim of her psychiatric illness while placing her actions in a transgenerational narrative. It recounts Barratt’s history of hearing voices, including ‘that of her great grandad, [which] told her to kill a woman called Stephanie Harris who had betrayed him to the Germans in the war’. The article is a dramatic account of the moments leading up to Barratt’s release from hospital and her act of murder. It ends with a completion of Barratt’s own psychotic narrative: ‘the psychiatrist let her out. And 48 hours after that, Barratt - screaming “Stephanie” - stabbed Emma to death’ (Oldfield and Hughes 1993). The cluster of articles surrounding Barratt’s case does not cohere around a single version of the psychiatric subject. Nor do they come together to create a clear emotional framework for understanding and consuming Barratt’s story. All the accounts of the Barratt case agree that the murder is an act of insanity. However, whether it is due to the culmination of the sufferings of a traumatised victim, the directionless, illegible violence of a ‘psycho killer’, or the tragic fall of a woman attempting an act of hallucinatory heroism is up for debate.

At a discursive level, the articles on Barratt present the psychiatric establishment as newly, doubly inadequate. No longer able to contain actual violence away from public spaces (much is made of the murder taking place in a shopping centre), it also no longer offers clear narratives through which the public might consume and understand these acts of violence. Throughout the 1990s, the *Daily Mirror* continued to report violent crimes committed by women in need of psychiatric help, in a manner that combined horrifying accounts of monstrous women with other stereotypes surrounding trauma, motherhood and unstable fantasies. Reporting on Karen McSweeney’s kidnap and murder of a 6-month-year-old baby in 1993 similarly contains more contradictions that would usually be held in a single tabloid article. The *Daily Mirror* report describes McSweeney as a ‘pathological liar’ who could not ‘distinguish fantasy from reality’ and was allowed to walk free, while also tracing the development of McSweeney’s delusions to the trauma of having a miscarriage (Ungoed-Thomas 1993). Female perpetrators of pathologised violence present the readers with a quandary in these accounts, and no final decision is made as to the causes of their actions. As objects of feeling, they are deeply emotionally ambivalent.

I argued earlier that the tabloid previously acted as a Foucauldian cage, allowing its readership to observe curated spectacles of madness without fear of contagion. Here we see that the cage has opened, and forms of madness that were not previously available to the public eye reveal themselves as the story-tellers struggle to create narrative frameworks that present psychiatric subjects in these new conditions. As Barbara Taylor highlights, female violence has long created problems for historically gendered understandings of mental illness:

Female lunacy is disreputable. […] Femininity had always been perceived as having a pathological element, embodied in such familiar figures as the breast-heaving hysteric and the wispy neurasthenic. These figures conform neatly to womanly stereotypes. But the noisy, disinhibited, disrupted madwoman is a perversion of nature, an anti-woman, especially when she is a mother (Taylor 2015, 170).

The monstrous ‘antiwoman’ emerges as an additional trope in these articles, negotiating column inches with genderless representations of murderers and the victim trope. The emerging popular obsession with the violent antiwoman in the 1990s speaks to a wider crisis of representation surrounding psychiatric subjects. In the 5 years following the passing of the Community Care Act, tabloid stories scrabble to describe what it means to suffer from mental distress in the absence of the institutions and hierarchies which have historically regulated supposedly pathological behaviours.

## Conclusion: objects of feeling

Tabloid newspapers produce and circulate objects of feeling for their readerships. By repeatedly framing objects in similar narratives, they come to stand in metonymically for entire structures or populations. Designed to create intense feelings of anger, hatred or excitement, these stories press onto the bodies of their readers, so that we feel ‘the ‘press’ of (their) impression’ (Ahmed 2004, 6). It is this insistent affective pressing which Philo has found to have the most powerful impact on readers, leading, for example, to readers working in mental health settings to feel afraid of their own patients without any other reason to do so (Happer and Philo 2013; Philo 1996). In this article, I have suggested that asylums and psychiatric hospitals functioned as important regulatory objects of feeling in tabloids until the start of the community care era. In the following 5 years, the asylum still has a presence in tabloids as the nostalgic and disciplinary solution to community care. The asylum walls gain greater emotional significance at this point even as they are revealed to be a fantasy.

Over the next decade, a new set of emotive and negative stereotypes would emerge in reporting on those with mental illness. Following the 2008 economic crash, mental health service users were met with the accusation of ‘scrounging’. France Ryan has described how disabled people and those with mental illness have been subject to severe deprivation under austerity policies, with the tabloid press which created emotive narratives to justify and encourage this cruelty and social neglect (Ryan 2019, 4). Both Ahmed’s and Ryan’s work act as an important reminder that emotions are not private. Rather they are circulated, shared and magnified in public spaces. In doing so, they shape the social and political structures in which decisions are made about what kind of care is made available and to whom.

## Data Availability

Data sharing is not applicable as no datasets were generated and/or analysed for this study. All articles analysed are publicly available in the British Library Newsroom.
